# Changes in Mitochondria-Related Gene Expression upon Acupuncture at LR3 in the D-Galactosamine-Induced Liver Damage Rat Model

**DOI:** 10.1155/2022/3294273

**Published:** 2022-06-29

**Authors:** Yu-Mi Lee, Dong-Hee Choi, Min-Woo Cheon, Jae Gwan Kim, Jeong-Sang Kim, Myung-Geun Shin, Hye-Ran Kim, Daehwan Youn

**Affiliations:** ^1^Department of Biomedical Science and Engineering, Institute of Integrated Technology, Gwangju Institute of Science and Technology(GIST), Gwangju, Republic of Korea; ^2^Department of Korean Medicine, School of Dongshin University, Naju, Jeollanam-do 58245, Republic of Korea; ^3^Department of Health Administration, Dongshin University, Naju, Jeollanam-do 58245, Republic of Korea; ^4^Department of Laboratory Medicine, Chonnam National University Medical School and Chonnam National University Hwasun Hospital, Hwasun, Republic of Korea

## Abstract

Hepatic diseases, such as hepatonecrosis, hepatitis, and hepatocirrhosis, are associated with mitochondrial dysfunction and increased reactive oxygen species generation and inflammation, ultimately leading to liver failure. In this study, we examined if acupuncture at LR3 can affect mitochondria-related gene expression in a liver damage model of experimentally induced acute liver failure (ALF). ALF was induced by the intraperitoneal injection of D-galactosamine (D-GalN) in experimental rats, who then received either sham (ALF), manual acupuncture (MA), electroacupuncture (EA), or silymarin (PC, positive control) treatment. Liver tissues were extracted from experimental and untreated control rats for histopathological analysis and expression profiling of genes involved in mitochondrial function. Of the 168 mitochondria-related genes profiled, two genes belonging to the solute-carrier transporter family (*Slc25a15* and *Slc25a25*) and *Ndufb7* were upregulated. Gamma-glutamylcysteine synthetase was more downregulated in MA than ALF. Furthermore, MA reversed D-GalN-induced inflammatory cell infiltration, destruction of hepatic cell plates, and increase in the levels of the proinflammatory cytokine TNF-*α*. MA at LR3 can reduce the risk of D-GalN-induced ALF by inducing the expression of metabolic and inflammation-related genes and regulating proinflammatory factor production in hepatic mitochondria.

## 1. Introduction

Acupuncture is one of the most widely used treatment methods in traditional Asian medicine for preventing and relieving the symptoms of acute and chronic pathophysiological conditions owing to its efficacy and safety [1–3]. Animal model studies have suggested its therapeutic value in liver disease. Liu et al. [4] reported that electroacupuncture (EA) at PC6 mitigated endotoxin-induced liver dysfunction in rats. Yim et al. [5] reported that acupuncture at GB34 reduced CCl_4_-induced liver toxicity and protected liver function. Moreover, EA at LR3 and TE4 was reported to prevent experimental acute liver failure (ALF) in rats [6], and acupuncture at LR3 prevented hepatocellular apoptosis [7].

ALF may be induced by drugs, viruses, and autoimmune infections [8], frequently leading to rapidly advancing multiorgan failure [9]. The mortality rate in ALF is high despite treatment, and patients may require a liver transplant for survival [10]. Mitochondrial dysfunction is a major contributor to hepatocellular injury in ALF [11]. Mitochondrial (mt) DNA damage causes dysfunctions in the mitochondrial respiratory chain and tricarboxylic acid cycle by decreasing mitochondrial transcription and inhibiting mitochondrial protein synthesis, inducing cell dysfunction or necrosis [11, 12].

Several studies have demonstrated the beneficial effects of acupuncture on mitochondrial function, including increased cytochrome *c* oxidase (complex IV) activity following acupuncture at LR3 [13]. Li et al. [14] reported that acupuncture significantly improved mitochondrial bioenergetic parameters, such as respiratory control rates and membrane potential, and prevented cognitive deficits associated with hippocampal mitochondrial dysfunction. Wang et al. [15] reported that EA treatment at CV4 and ST36 and manual acupuncture (MA) at GV20 reduced hepatic mitochondrial oxygen consumption in aging animals, leading to an improved respiratory control rate and phosphorus/oxygen ratio. However, few studies have examined the effects of acupuncture on liver disease.

In this study, we examined the effects of acupuncture at LR3 on mitochondria-related gene expression in a liver damage model of experimentally induced ALF and evaluated the underlying mechanisms.

## 2. Materials and Methods

### 2.1. Animals

Pathogen-free male Wistar rats (150–180 g) were purchased from SamTako Bio (Osan, Korea) and housed under controlled temperature (24-25°C) and humidity (40%–60%) and a 12 h-12 h dark-light cycle with ad libitum access to filtered tap water and food (Pellet, GMO, Korea). All animal care and experimental protocols were approved by the College Animal Management and Use Commission of Dongshin University (approval number: DSU-2019-05-02). All efforts were made to minimize animal suffering.

### 2.2. Induction of ALF and Grouping

Twenty-five male Wistar rats were randomly divided into five groups, including four experimental groups (ALF, positive control (PC), MA, and EA) and one untreated control group (control). Experimental animals were first injected with D-GalN (Sigma, St. Louis, USA; 700 mg/kg, intraperitoneal injection; i.p.) to induce ALF [16] and then given sham treatment (ALF), acupuncture treatment (MA or EA performed once every 3 d, for a total of seven administrations), or silymarin (Sigma, St. Louis, USA; 700 mg/kg, p.o.) 6 h after ALF induction as PC. All rats were euthanized by anesthesia overdose 24 h after ALF was induced.

### 2.3. Acupuncture Stimulation

Acupuncture was conducted as described by Choi et al. [13] at LR3 following the standard method [17]. The rats were subjected to inhalation anesthesia (following induction with 5% isoflurane, anesthesia was maintained at a concentration of 2%). Settings of the EA apparatus were adjusted to 3 V and 10 Hz, and a needle was placed into the muscle layer at the acupoint at a depth of 2-3 mm. The positive charge was introduced at the right acupoint and the negative charge at the left acupoint. Stimulation was performed for 5 min.

### 2.4. RNA Isolation

The liver tissue was washed three times with PBS, cut into 50 mg samples, and lysed with 1 mL of TRIzol reagent (Thermo Fisher Scientific, Waltham, USA). Whole-cell RNA was extracted using a standard protocol [18], and the yield was measured using the nanodrop spectrophotometer (Thermo Fisher Scientific, Waltham, USA). Reverse transcription was performed using the RT^2^ First Strand Kit (Qiagen, Valencia, USA), according to the manufacturer's instructions.

### 2.5. Quantitative RT-PCR Array for Mitochondria-Related Gene Expression

The Rat Mitochondria and Mitochondrial Metabolism RT^2^ Profiler PCR arrays (Qiagen, Valencia, USA) were used for quantifying real-time PCR expression of 164 mitochondrial genes (84 per array). Real-time PCR for the RT^2^ profiler PCR array was performed using the RT^2^ SYBR Green qPCR MasterMix and oligo-dT primers (Qiagen, Valencia, USA).

### 2.6. Tumor Necrosis Factor-*α* Levels

Tumor necrosis factor-*α* (TNF-*α*) was quantified using a kit (Thermo Fisher Scientific, Waltham, USA) in plasma samples acquired 24 h after ALF induction using a microplate-based spectrophotometer (Biochrom, Cambridge, UK), according to an automated procedure [19].

### 2.7. Histopathological Analysis and Immunohistochemistry

Liver tissues were fixed in Bouin's solution (Sigma, St. Louis, USA), embedded in paraffin, sectioned at 6 *μ*m, and stained using hematoxylin and eosin (H&E; Sigma, St. Louis, USA) and Masson's trichrome stain (Trichome stain kit; ScyTek Laboratories, West Logan, USA) using standard protocols [20, 21]. Nuclear counterstaining was performed using hematoxylin, and the samples were examined using light microscopy (Nikon, Tokyo, Japan). For *Slc25a15* immunostaining, the samples were incubated first with 1 : 300 dilutions of the anti-*Slc25a15* antibody (Abcam, Cambridge, UK) and then with a biotinylated anti-mouse IgG (Vectastain ABC Kit; Vector Labs, Burlingame, USA). The sections were incubated with the avidin–biotin–peroxidase complex (Vectastain ABC Kit; Vector Labs, Burlingame, USA) and DAB. The Celleste image analysis software (Thermo Fisher Scientific, Waltham, USA) was used to count the number of immunoreactive cells.

### 2.8. Data Analysis

#### 2.8.1. Statistical Analyses for Arrays

Real-time PCR data were analyzed through the ΔΔCt method using the Qiagen Gene Globe Data Analysis Center portal (https://www.qiagen.com/us/shop/genesand-pathways/data-analysis-center-overview-page). Control wells of real-time PCR arrays detect genomic contamination and serve as reverse transcription and positive PCR controls. The following five reference genes were used for data normalization: beta-actin, lactate dehydrogenase A, ribosomal protein large P1, beta-2 microglobulin, and hypoxanthine phosphoribosyltransferase 1. Genes with an absolute fold-change in expression > 2 at a *p* < 0.05 relative to other groups were considered differentially expressed and selected for comparative analysis.

#### 2.8.2. Statistical Analyses for ELISA and IHC

The GraphPad Prism 8.4.1 Software (GraphPad Software, San Diego, USA) was used for computational and statistical analyses. Tukey's multiple comparison test was used to estimate the normality of all results. The results are expressed as mean ± SD. A *p* value of < 0.05 was considered statistically significant.

## 3. Results

### 3.1. Manual Acupuncture Reversed the Liver Damage-Associated Dysregulation of Mitochondrial Genes

To evaluate whether acupuncture treatment had an effect on the mitochondrial gene expression profiles of rat liver, tissues from ALF, control, PC, MA, and EA were analyzed to identify 84 mitochondria genes using the RT^2^ profiler PCR arrays test.

**Figure 1:**
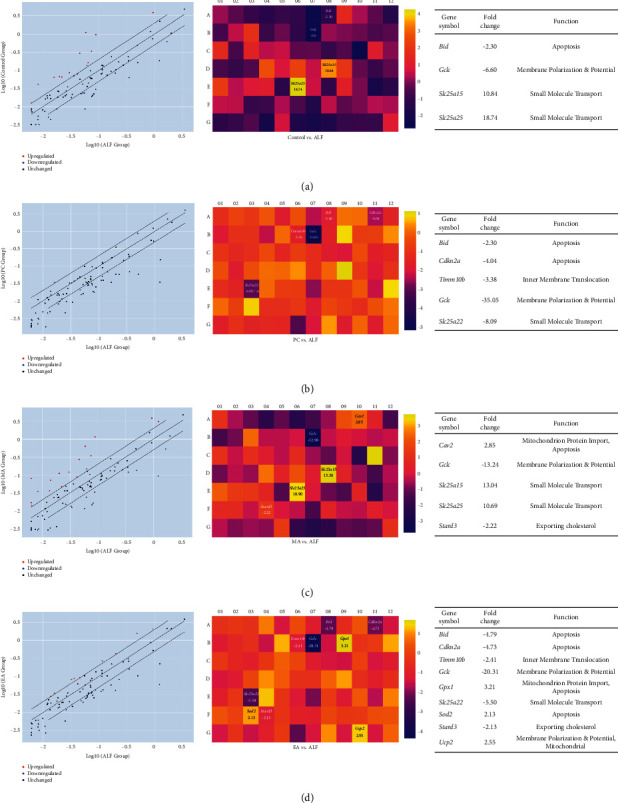
Expression profiles of mitochondria-related genes following chemically induced acute liver failure (ALF) were measured using a real-time PCR array. Differentially expressed genes are defined relative to ALF. (a) ALF vs. control. (b) ALF vs. silymarin (PC, positive control). (c) ALF vs. manual acupuncture (MA). (d) ALF vs. electroacupuncture (EA). The panels on the left show scatterplots (*p* < 0.05 vs. ALF); those in the middle show heatmaps of differential expression; and the ones on the right show tables of fold regulation.

**Figure 2:**
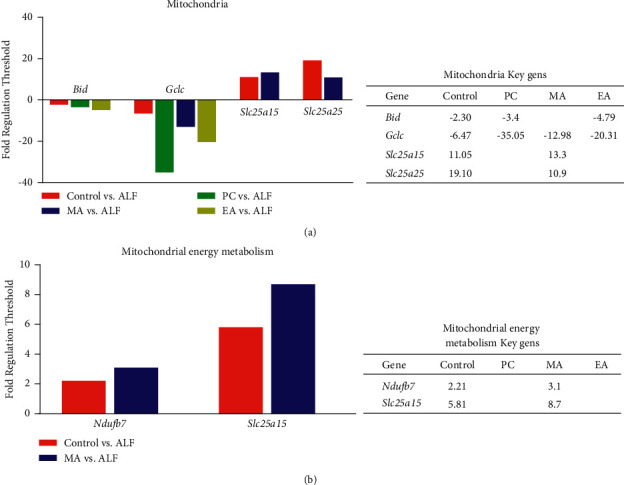
Fold-change in the expression levels of genes included in the (a) mitochondrial and (b) mitochondrial energy metabolism real-time PCR arrays. ALF, acute liver failure and no treatment; control, no induction and no treatment; PC, silymarin treatment; MA, manual acupuncture treatment; EA, electroacupuncture treatment.

A total of 68 genes showed statistically significant changes in comparison with those in ALF (Supplementary Table 1). In the control group, 29 genes (12 up and 17 downregulated) showed changes compared with ALF; *Bid*, BH3-interacting domain death agonist (*Bid*), and gamma-glutamylcysteine synthetase (*Gclc*) were downregulated, and *Slc25a15* and *Slc25a25* were significantly upregulated. In PC, 44 genes (1 up and 43 downregulated) showed changes compared with ALF; *Bid* and *Gclc* were downregulated. In MA, 37 genes (16 up and 21 downregulated) showed changes compared with ALF; *Gclc* was downregulated; *Slc25a15* and *Slc25a25* were upregulated. In EA, 43 genes (9 up and 34 downregulated) showed changes compared with ALF; *Bid* and *Gclc* were downregulated (Figures 1 and 2(a)).

To explore whether acupuncture treatment had any effect on expression profiles of genes involved in the mitochondrial energy metabolism of rat liver, tissues from ALF, control, PC, MA, and EA were analyzed using the RT^2^ profiler PCR arrays to identify 84 mitochondrial energy metabolism genes.

A total of 38 genes showed significant changes when compared with those in ALF (Supplementary Table 2).

**Figure 3:**
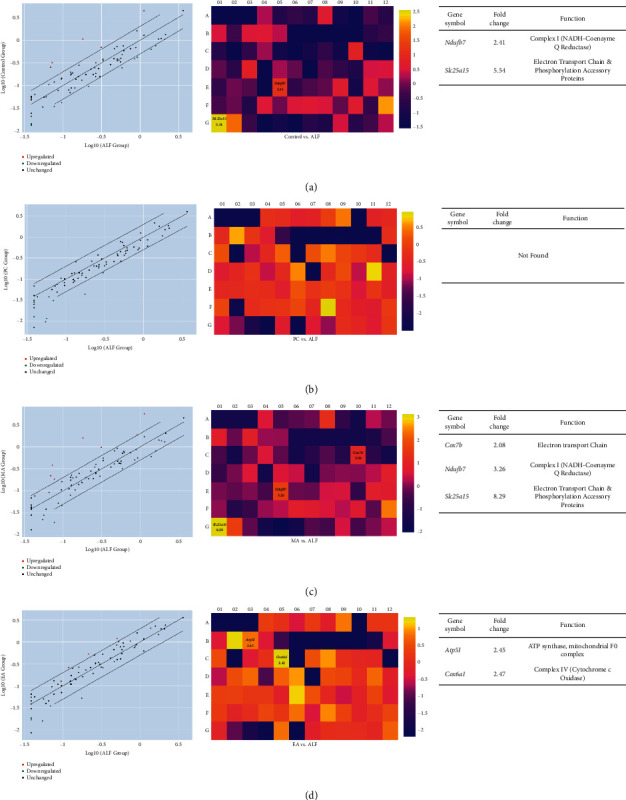
Expression profiles of genes related to the mitochondrial metabolism following chemically induced acute liver failure (ALF), measured using real-time PCR arrays. Differentially expressed genes are defined relative to ALF. (a) ALF vs. control. (b) ALF vs. silymarin (PC, positive control). (c) ALF vs. manual acupuncture (MA). (d) ALF vs. electroacupuncture (EA). The panels on the left show scatterplots (*p* < 0.05 vs. ALF), panels in the middle show heatmaps of differential expression, and those on the right show tables of fold regulation.

In the control, 28 genes (4 up and 24 downregulated) showed changes compared with ALF; *Ndufb7* and *Slc25a15* were upregulated. In PC, 21 genes (all downregulated) showed changes compared with ALF. However, there were no markers in PC among these genes. In MA, 21 genes (2 up and 19 downregulated) showed changes compared with ALF; *Ndufb7* and *Slc25a15* were upregulated. In EA, 27 genes (7 up and 20 downregulated) showed changes compared with ALF. However, there were no markers in EA among these genes (Figures 3 and 2(b)).

Therefore, results from the evaluation of mitochondria and mitochondrial energy metabolism genes revealed that *Slc25a15* is a key gene in MA.

**Figure 4:**
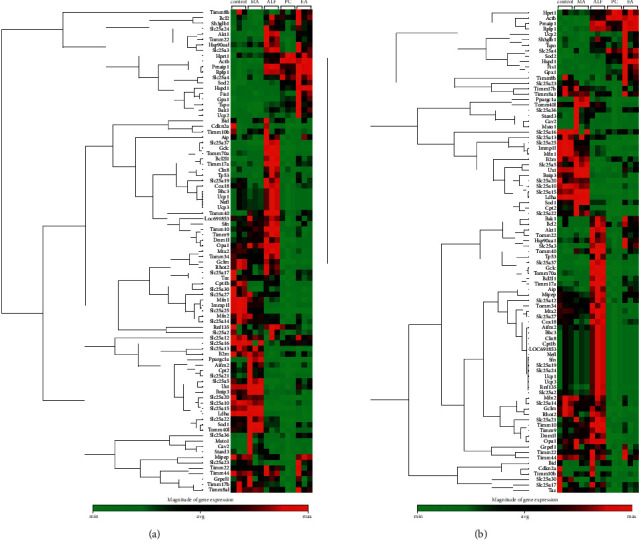
Clustergram of the (a) mitochondrial and (b) mitochondrial energy metabolism real-time PCR array results. The clustergram shows the nonsupervised hierarchical clustering dendrogram and heatmap identifying coregulated genes across groups. Red means higher expression and green means lower expression.

Notably, differences in expression between MA and ALF resembled patterns between control and ALF (Figure 4), suggesting that MA reversed ALF-induced expression changes. We speculate that the reversal of ALF-associated expression changes by MA could help protect mitochondrial function, thereby reducing inflammation and tissue degeneration.

### 3.2. Manual Acupuncture Protects against TNF-*α*-Mediated Hepatic Tissue Damage by Upregulating Slc25a15

**Figure 5:**
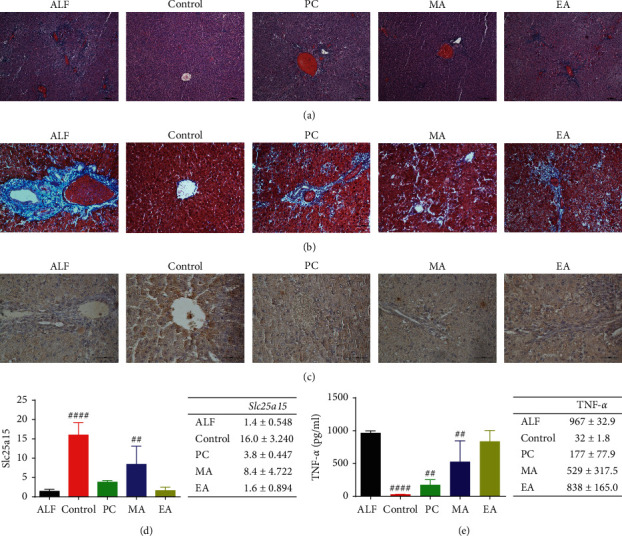
Representative photomicrographs of liver histology. (a) Hematoxylin and eosin (magnification: 100x) and (b) Masson's trichrome (magnification: 200x) staining. (c), (d) Immunohistochemical observation of *Slc25a15* in the liver tissue. Data are presented as mean ± SD. ^####^*P* < 0.0001, ^##^*p* < 0.01 vs. ALF (magnification: 400x). (e) TNF-*α* concentration in blood samples obtained 24 h after ALF induction. Data are presented as mean ± SD. ^####^*P* < 0.0001, ^##^*p* < 0.01 vs. ALF.

Results from H&E staining revealed higher inflammatory infiltration, congestion, and tissue collapse in the liver tissue of ALF compared with the liver tissue in the control. All treatment groups showed lesser inflammatory infiltration and tissue damage than ALF. Notably, lesser inflammatory infiltration and tissue damage were observed in MA than in other treatment groups (Figure 5(a)).

Results from Masson's trichrome staining revealed that ALF showed an increase in collagen fiber deposition in the liver tissue, with each treatment group showing lesser collagen fiber deposition than ALF. In MA, lesser fibrotic deposition and congestion were observed than in other treatment groups (Figure 5(b)).

Results from immunohistostaining revealed the distribution of *Slc25a15*, a key gene of the mitochondria and mitochondrial energy metabolism. High expression was observed in the liver in control and MA groups (Figures 5(c) and 5(d)).

Furthermore, consistent with the potential protective effect of MA-induced changes in gene expression, the increased expression of proinflammatory TNF-*α* in ALF compared with the control (*p* < 0.0001) was reversed in PC and MA (*p* < 0.01) (Figure 5(e)).

Notes: ALF, acute liver failure and no treatment; control, no induction and no treatment; PC, silymarin treatment; MA, manual acupuncture treatment; EA, electroacupuncture treatment.

## 4. Discussion

Mitochondria dysfunction is a major driver of cellular inflammatory responses and apoptosis and thus contributes to many pathological conditions [22]. Mitochondrial factors contributing to cell death include cytochrome c, endonuclease G apoptosis-inducing factor, Smac/DIABLO, HtrA2/OMI, and adenylate kinase 2 [23]. Mutations in mtDNA caused by mitochondrial dysfunction were reported to contribute to the pathogenesis of chronic inflammatory diseases, including neuromuscular and neurodegenerative disorders [24]. However, little is known regarding the contributions of mitochondrial gene dysregulation in disease or the potential protective efficacy of reversing this dysregulation. Through this study, we demonstrated the association between liver damage and dysregulation of multiple mitochondria-associated genes using a model of experimentally induced ALF and that acupuncture can be used to reverse this dysregulation and attenuate early degeneration and immune cell infiltration of liver tissue.

D-GalN induces ALF by triggering ROS production, followed by hepatic inflammation and apoptosis [25], which are pathogenic processes implicated in many liver diseases. Thus, D-GalN-induced ALF is a widely used model of hepatic injury [26]. D-GalN reduces mitochondrial membrane fluidity and the activity of mitochondrial enzymes and ion transporters, resulting in metabolic failure and ultimately hepatic failure [27].

Silymarin is a polyphenolic flavonoid derived from milk thistle (*Silybum marianum*), and it is used as a standard agent that exhibited significant hepatoprotective activity in addition to anti-inflammatory, cytoprotective, and anticarcinogenic effects against D-GalN [28].

In this study, we screened 168 genes, related to the mitochondria and mitochondrial energy metabolism, to analyze the effect of acupuncture at LR3 on genes that regulate the reversal of liver damage in a rat model.

*Bid* was cloned based on its ability to interact with both Bcl-2 and Bax. Bid only contains the BH3 domain, which is required for its interaction with the Bcl-2 family proteins and for its proapoptotic activity [29].

In this study, *Bid* expression was downregulated in all experimental groups, except in MA, compared with ALF. Unlike MA, EA affected *Bid* expression.

*Gclc* catalyzes the first rate-limiting step of glutathione synthesis and encodes a catalytic and light regulatory subunit. *Gclc* overexpression was reported to inhibit endoplasmic reticulum stress and the downstream inflammatory factor [30].

In this study, *Gclc* expression was downregulated in all experimental groups compared with the ALF. MA and EA may facilitate a mechanism for the maintenance of cellular GSH homeostasis.

*Slc25a25* belongs to a family of calcium-binding mitochondrial carriers. The protein encoded by *Slc25a25* binds PGC-1a, which acts as an ATP carrier. *Slc25a25* is also involved in the regulation of glucagon, the deficiency or depletion of which can reduce glucose-dependent ATP production [31]. In this study, we found that the restoration of normal expression levels by MA can help maintain the ATP supply required to mitigate the effects of D-GalN.

*Ndufb7* contributes to the regulation of complex I (NADH-coenzyme Q reductase) functions. The absence or deficiency of *Ndufb7* induces complex I defects. Rescue of function with *Ndufb7* restores complex I activation [32]. In this study, we have shown that the restoration of *Ndufb7* expression may contribute to MA-mediated hepatoprotection by sustaining complex I activity.

*Slc25a15* is a member of the mitochondrial carrier family and provides instructions for making a protein called mitochondrial ornithine transporter 1. The encoded protein transports ornithine across the inner mitochondrial membrane from the cytosol to the mitochondrial matrix. The protein is an essential component of the urea cycle and functions in ammonium detoxification and arginine biosynthesis. *Slc25a15* was reported to be associated with the involvement of ornithine in the brain energy metabolism homeostasis, cellular ATP transfer, and inflammation. [33].

In this study, *Slc25a15* expression was upregulated in MA compared with ALF in both mitochondrial and mitochondrial energy metabolism genes. MA most likely contributes to hepatoprotective effects against D-GalN by regulating the expression of proinflammatory genes.

Several mitochondrial carriers, such as *Slc25a15*, are involved in the inflammatory process [34]. Therefore, we conducted histopathological analysis and measured blood concentrations of the proinflammatory factor TNF-*α*. MA reversed D-GalN-induced upregulation of TNF-*α*, suggesting that MA protects against hepatic damage by suppressing system inflammation. Acupuncture at ST36, CV4, and KI1 was reported to reduce inflammatory factors, such as TNF-*α*, and inflammatory cell infiltration in a nonalcoholic fatty liver disease model [35]. Moreover, MA at ST36 regulated inflammatory factors in hepatitis models [36].

Liver damage was also assessed by using H&E with Masson's trichrome staining to evaluate the disruption of the cellular structure in the liver and fibrotic septa [37].

H&E staining revealed that inflammatory cell infiltration, destruction of hepatic cell plates, and structural disruption of hepatic lobules observed in ALF was mitigated by MA, and Masson's trichrome staining showed that tissue damage and fibrosis observed in ALF were mitigated by MA.

The histological observations demonstrate that the hepatoprotective effect of MA may result from the regulation of inflammatory factors.

To confirm *Slc25a15* expression, the immunohistochemical distribution of *Slc25a15* in liver tissue was observed and appeared to be similarly upregulated in control and MA in both mitochondria and mitochondria energy metabolism genes. We confirmed that the immunoreactivity of *Slc25a15* in control and MA increased compared with that in ALF, which was the same as the results of the RT^2^ profiler PCR array.

In summary, our results from screening gene expression profiles and histopathological, immunohistochemical, and proinflammatory mediator analyses demonstrated that the MA-induced reduction in TNF-*α* reflects a reduction in hepatic inflammation due to the preservation of mitochondrial function, which in turn results from the restoration of *Slc25a15* expression levels in D-GalN-induced liver damage.

Limitations of this study include the lack of basic liver function tests, observations of inflammation-linked mechanisms or proteins related to inflammation, and observations during the recovery period following hepatic injury. Further investigations are required to confirm the hepatoprotective mechanisms of MA through cross-validation of mitochondrial genes and inflammation-related proteins.

## 5. Conclusions

Reduction of inflammation in liver tissue and recovery of the histological structure were observed. The MA group showed recovery compared with other experimental groups. A reduction in the TNF-*α* level was observed after this type of acupuncture stimulation. The recovery effect was linked to changes in the expression of *Slc25a15*, which is one of the 168 genes in the mitochondria.

Collectively, these results suggest that acupuncture can reduce liver injury by upregulating genes associated with the mitochondria and mitochondrial energy metabolism, thereby reducing inflammation and hepatic cell apoptosis.

## Data Availability

The datasets used and analyzed during this study are available from the corresponding author upon request.

## Supplementary Materials

Supplementary Materials Information on profiling genes that showed significant changes among a total of 164 mitochondrial-related genes are given in supplementary tables. Supplementary Table 1: profiling of 84 mitochondria genes related to membrane polarization and potential, mitochondrial transport, small molecule transport, targeting proteins to mitochondria, mitochondrion protein import, outer membrane translocation, inner membrane translocation, mitochondrial fission and fusion, mitochondrial localization, and apoptosis obtained identified 68 differentially expressed genes relative to the ALF group. Supplementary Table 2: profiling of 84 genes related to complex I, complex II, complex III, complex IV, electron transport chain, and oxidative phosphorylation identified 38 differentially expressed genes relative to the ALF group.
