# Supplementary material for: Changes in Mitochondria-Related Gene Expression upon Acupuncture at LR3 in the D-Galactosamine-Induced Liver Damage Rat Model
Source: Evid Based Complement Alternat Med. 2022 Jun 29;2022:3294273. doi: 10.1155/2022/3294273 (PMC9345726; doi:10.1155/2022/3294273)
Supplement: Supplementary Materials — Information on profiling genes that showed significant changes among a total of 164 mitochondrial-related genes are given in supplementary tables. Supplementary Table 1: profiling of 84 mitochondria genes related to membrane polarization and potential, mitochondrial transport, small molecule transport, targeting proteins to mitochondria, mitochondrion protein import, outer membrane translocation, inner membrane translocation, mitochondrial fission and fusion, mitochondrial localization, and apoptosis obtained identified 68 differentially expressed genes relative to the ALF group. Supplementary Table 2: profiling of 84 genes related to complex I, complex II, complex III, complex IV, electron transport chain, and oxidative phosphorylation identified 38 differentially expressed genes relative to the ALF group. [file 3294273.f1.zip › 3294273.f1/SUPPLEMENTARY DESCRIPTION.docx]

SUPPLEMENTARY DESCRIPTION

The ARRIVE guidelines 2.0: author checklist

Refer to the 'Supplementary tables' file for information on profiling genes that showed significant changes among a total of 164 mitochondrial-related genes.

Supplementary Table 1 - Profiling of 84 mitochondria genes related to membrane polarization&potential, mitochondrial transport, small molecule transport, targeting proteins to mitochondria, mitochondrion protein import, outer membrane translocation, inner membrane translocation, mitochondrial fission&fusion, mitochondrial localization, and apoptosis were obtained identified another 68 differentially expressed genes relative to the ALF group.

Supplementary Table 2 - Profiling of 84 genes related to Complex I, Complex II, Complex III, Complex IV, electron transport chain, and oxidative phosphorylation identified another 38 differentially expressed genes relative to the ALF group.
