# Supplementary material for: Changes in Mitochondria-Related Gene Expression upon Acupuncture at LR3 in the D-Galactosamine-Induced Liver Damage Rat Model
Source: Evid Based Complement Alternat Med. 2022 Jun 29;2022:3294273. doi: 10.1155/2022/3294273 (PMC9345726; doi:10.1155/2022/3294273)
Supplement: Supplementary Materials — Information on profiling genes that showed significant changes among a total of 164 mitochondrial-related genes are given in supplementary tables. Supplementary Table 1: profiling of 84 mitochondria genes related to membrane polarization and potential, mitochondrial transport, small molecule transport, targeting proteins to mitochondria, mitochondrion protein import, outer membrane translocation, inner membrane translocation, mitochondrial fission and fusion, mitochondrial localization, and apoptosis obtained identified 68 differentially expressed genes relative to the ALF group. Supplementary Table 2: profiling of 84 genes related to complex I, complex II, complex III, complex IV, electron transport chain, and oxidative phosphorylation identified 38 differentially expressed genes relative to the ALF group. [file 3294273.f1.zip › 3294273.f1/Supplementary tables.docx]

| Supplementary Table 1 – Genes that showed significant differences among 84 mitochondrial genes. | | | | | | | | | | | |
| --- | --- | --- | --- | --- | --- | --- | --- | --- | --- | --- | --- |
| Gene | Con | PC | MA | EA | Function | Gene | Con | PC | MA | EA | Function |
| Aifm2 | - | ↓ | ↑ | - | Apoptosis | Slc25a10 | ↑ | - | ↑ | ↑ | Small Molecule Transport |
| Akt1 | - | - | ↓ | - | Apoptosis | Slc25a13 | ↑ | - | ↑ | - | Small Molecule Transport |
| Bak1 | ↓ | - | ↓ | - | Membrane Polarization & Potential, Mitochondrial Transport, Apoptosis | Slc25a14 | - | ↓ | - | - | Small Molecule Transport |
| Bbc3 | - | ↓ | ↓ | ↓ | Apoptosis | Slc25a15 | ↑ | - | ↑ | - | Small Molecule Transport |
| Bcl2 | ↓ | - | ↓ | - | Membrane Polarization & Potential, Mitochondrial Transport, Apoptosis | Slc25a16 | ↑ | ↑ | - | ↑ | Small Molecule Transport |
| Bcl2l1 | ↓ | ↓ | ↓ | - | Membrane Polarization & Potential, Mitochondrial Transport, Apoptosis | Slc25a19 | - | ↓ | ↓ | ↓ | Small Molecule Transport |
| Bid | ↓ | ↓ | - | ↓ | Apoptosis | Slc25a20 | ↑ | ↓ | ↑ | ↓ | Small Molecule Transport |
| Bnip3 | ↑ | - | ↑ | - | Membrane Polarization & Potential, Mitochondrial Transport, Apoptosis | Slc25a2 | - | ↓ | - | - | Small Molecule Transport |
| Cav2 | - | - | ↑ | - | Mitochondrion Protein Import | Slc25a21 | - | ↓ | ↑ | - | Small Molecule Transport |
| Cdkn2a | - | ↓ | - | ↓ | Apoptosis | Slc25a22 | - | ↓ | ↑ | ↓ | Small Molecule Transport |
| Cln8 | ↓ | ↓ | ↓ | ↓ | Mitochondrion Protein Import | Slc25a23 | - | - | - | ↑ | Small Molecule Transport |
| Cox18 | - | ↓ | ↓ | ↓ | Mitochondrion Protein Import, Mitochondrial Fission & Fusion | Slc25a24 | ↓ | - | ↓ | - | Small Molecule Transport |
| Cpt1b | - | ↓ | - | - | Mitochondrial Transport | Slc25a25 | ↑ | - | ↑ | ↓ | Small Molecule Transport |
| Cpt2 | - | ↓ | ↑ | - | Mitochondrial Transport | Slc25a27 | - | ↓ | - | ↓ | Small Molecule Transport |
| Dnm1l | - | ↓ | - | ↓ | Mitochondrial Localization, Apoptosis | Slc25a30 | ↑ | - | ↓ | ↓ | Small Molecule Transport |
| Timm10b | ↓ | ↓ | - | ↓ | Inner Membrane Translocation | Slc25a37 | ↓ | ↓ | ↓ | ↓ | Small Molecule Transport |
| Gclc | ↓ | ↓ | ↓ | ↓ | Membrane Polarization & Potential | Slc25a4 | ↓ | - | - | - | Small Molecule Transport |
| Gclm | - | ↓ | - | ↓ | Membrane Polarization & Potential | Sod2 | - | - | - | ↑ | Apoptosis |
| LOC691853 | - | ↓ | - | - | Mitochondrion Protein Import | Stard3 | - | ↓ | ↑ | ↓ | Mitochondrial Transport |
| Fis1 | - | - | - | ↑ | Mitochondrial Fission & Fusion | Taz | - | ↓ | - | - | Inner Membrane Translocation |
| Gpx1 | - | - | - | ↑ | Mitochondrion Protein Import, Apoptosis | Timm17a | ↓ | ↓ | ↓ | ↓ | Inner Membrane Translocation |
| Hspd1 | - | - | - | ↑ | Mitochondrial Transport, Targeting Proteins to Mitochondria, Mitochondrion Protein Import | Timm9 | ↓ | ↓ | - | ↓ | Inner Membrane Translocation |
| Immp1l | ↑ | - | ↑ | - | Inner Membrane Translocation | Timm44 | - | ↓ | - | - | Inner Membrane Translocation |
| Mfn1 | ↑ | ↓ | - | ↓ | Mitochondrial Fission & Fusion | Tomm22 | - | ↓ | - | - | Outer Membrane Translocation |
| Mfn2 | - | - | - | ↓ | Mitochondrial Transport, Targeting Proteins to Mitochondria, Mitochondrial Fission & Fusion, Mitochondrial localization | Tomm34 | - | ↓ | - | ↓ | Outer Membrane Translocation |
| Mipep | - | ↓ | - | - | Mitochondrial Transport, Targeting Proteins to Mitochondria | Tomm40 | ↓ | ↓ | - | ↓ | Outer Membrane Translocation |
| Mtx2 | - | ↓ | - | - | Mitochondrial Transport | Tomm40l | - | ↓ | ↑ | - | Outer Membrane Translocation |
| Nefl | - | ↓ | ↓ | ↓ | Mitochondrial Localization | Tomm70a | ↓ | ↓ | ↓ | ↓ | Outer Membrane Translocation |
| Opa1 | - | ↓ | - | ↓ | Inner Membrane Translocation | Tp53 | ↓ | ↓ | ↓ | ↓ | Membrane Polarization & Potential, Mitochondrial Transport, Apoptosis |
| Ppargc1a | ↑ | ↓ | ↑ | ↓ | Mitochondrion Protein Import | Tspo | ↓ | - | ↓ | ↑ | Mitochondrial Transport, Targeting Proteins to Mitochondria |
| Pmaip1 | ↓ | - | ↓ | - | Apoptosis | Ucp1 | - | ↓ | ↓ | ↓ | Membrane Polarization & Potential, Mitochondrial Transport, Apoptosis |
| Rhot2 | - | ↓ | - | ↓ | Mitochondrial Fission & Fusion | Ucp2 | - | - | ↓ | ↑ | Membrane Polarization & Potential, Mitochondrial Transport, Apoptosis |
| Rnf135 | - | - | - | ↓ | Mitochondrial localization | Ucp3 | - | ↓ | ↓ | ↓ | Membrane Polarization & Potential, Mitochondrial Transpor |
| Sfn | - | ↓ | - | ↓ | Apoptosis | Uxt | - | - | ↑ | - | Mitochondrial localization |
|  |  |  |  |  |  |  |  |  |  |  |  |

| Supplementary Table 2 – Genes that showed significant differences among 84 mitochondrial metabolism genes. | | | | | | | | | | | |
| --- | --- | --- | --- | --- | --- | --- | --- | --- | --- | --- | --- |
| Gene | con | PC | MA | EA | Function | Gene | Con | PC | MA | EA | Function |
| Atp12a | ↓ | ↓ | ↓ | ↓ | ComplexⅤ(ATP Synthase) | Cox8a | ↓ | - | - | - | Complex Ⅳ(Cytochrome c Oxidase) |
| Atp4a | ↓ | ↓ | ↓ | ↓ | ComplexⅤ(ATP Synthase) | Cox8c | ↓ | ↓ | ↓ | ↓ | Complex Ⅳ(Cytochrome c Oxidase) |
| Atp4b | ↓ | ↓ | ↓ | ↓ | ComplexⅤ(ATP Synthase) | Cox15 | - | ↓ | ↓ | ↓ | Complex Ⅳ(Cytochrome c Oxidase) |
| Atp5f1 | - | - | ↑ | - | ComplexⅤ(ATP Synthase) | Ndufa10 | ↓ | - | ↓ | - | Complex Ⅰ(NADH-Coenzyme Q Reductase) |
| Atp5g2 | ↓ | - | - | - | ComplexⅤ(ATP Synthase) | Ndufa5 | ↓ | ↓ | ↓ | ↓ | Complex Ⅰ(NADH-Coenzyme Q Reductase) |
| Atp5g3 | ↓ | ↓ | ↓ | ↓ | ComplexⅤ(ATP Synthase) | Ndufa6 | ↓ | - | - | - | Complex Ⅰ(NADH-Coenzyme Q Reductase) |
| Atp5l | - | - | - | ↑ | ComplexⅤ(ATP Synthase) | Ndufb7 | ↑ | - | ↑ | - | Complex Ⅰ(NADH-Coenzyme Q Reductase) |
| Atp6v0a2 | - | ↓ | - | ↓ | ComplexⅤ(ATP Synthase | Ndufb8 | - | ↑ | - | - | Complex Ⅰ(NADH-Coenzyme Q Reductase) |
| Atp6v0d2 | ↓ | ↓ | ↓ | ↓ | ComplexⅤ(ATP Synthase) | Ndufs2 | ↓ | - | - | - | Complex Ⅰ(NADH-Coenzyme Q Reductase) |
| Atp6v1c2 | ↓ | ↓ | ↓ | ↓ | ComplexⅤ(ATP Synthase) | Ndufs7 | - | ↓ | ↓ | - | Complex Ⅰ(NADH-Coenzyme Q Reductase) |
| Atp6v1e2 | ↓ | ↓ | ↓ | ↓ | ComplexⅤ(ATP Synthase) | Ndufa10 | ↓ | - | - | ↓ | Complex Ⅰ(NADH-Coenzyme Q Reductase) |
| Atp6v1g3 | ↓ | ↓ | ↓ | ↓ | ComplexⅤ(ATP Synthase) | Slc25a10 | ↑ | - | ↑ | - | Electron Transport Chain & Phosphorylation Accessory Proteins |
| Bcs1l | ↓ | ↓ | ↓ | ↓ | ComplexⅢ(Coenzyme Q-Cytochrome c Reductase) | Slc25a15 | ↑ | - | ↑ | - | Electron Transport Chain & Phosphorylation Accessory Proteins |
| Cox4i2 | ↓ | ↓ | ↓ | ↓ | Complex Ⅳ(Cytochrome c Oxidase) | Slc25a20 | ↑ | ↓ | ↑ | - | Electron Transport Chain & Phosphorylation Accessory Proteins |
| Cox6a1 | ↓ | - | - | ↑ | Complex Ⅳ(Cytochrome c Oxidase) | Surf1 | - | ↓ | - | ↓ | Electron Transport Chain & Phosphorylation Accessory Proteins |
| Cox6a2 | ↓ | ↓ | ↓ | ↓ | Complex Ⅳ(Cytochrome c Oxidase) | Ucp1 | ↓ | ↓ | ↓ | ↓ | Electron Transport Chain & Phosphorylation Accessory Proteins |
| Cox6c | ↓ | - | - | - | Complex Ⅳ(Cytochrome c Oxidase) | Ucp2 | ↓ | - | ↓ | - | Electron Transport Chain & Phosphorylation Accessory Proteins |
| Cox7a2l | ↓ | - | ↓ | - | Complex Ⅳ(Cytochrome c Oxidase) | Ucp3 | ↓ | ↓ | ↓ | ↓ | Electron Transport Chain & Phosphorylation Accessory Proteins |
| Cox7b | - | - | ↑ | - | Complex Ⅳ(Cytochrome c Oxidase) | Uqcrc1 | - | ↓ | - |  | Complex Ⅲ(Coenzyme Q-Cytochrome Reductase) |
|  |  |  |  |  |  |  |  |  |  |  |  |
